# Case of Penetrating Gun-shot Wound of the Heart

**Published:** 1855-05

**Authors:** J. M. Carnochan


Case of penetrating gun-shot wound of the heart—life protracted for eleven days—bullet found buried and encysted in the substance of the heart. By J. M. Carnochan, M. D.—On the 27th of February, 1855, I was called in consultation to see William Poole, a young man aged 33 years, of unusually athletic form and muscular development, who had been wounded two days previously in an affray with fire-arms. He had received a bullet wound in the outer aspect of the right thigh, two inches above the upper border of the patella. The wound, however, which created alarm among his friends, was situated upon the anterior wall of the thorax, about three-quarters of an inch to the left of the mesial line, and about half an inch below a line drawn across the chest, from one nipple to the other. A bullet probe could be passed slantingly from right to left, along the track of the wound, for about an inch. At this depth, the probe was arrested, and it was not thought expedient to use force in making farther exploration. Poole received his wounds during a deliberate onslaught made on him by some five or six persons, armed with Colt's revolvers. The first ball took effect on the right thigh, and brought him to the ground. While thus prostrate, another assailant placed the muzzle of a pistol close to his chest, and discharged its contents. He immediately jumped up, and reeling towards a door, rested, as if stunned, against it for support, during some minutes. He then fell, exclaimed that he was dying, and remained senseless, cold, almost pulseless, and apparently moribund, for about four hours. From this condition he rallied, and became so free from the usual symptoms of severe injury, that his medical adviser, Dr. Putnam, considered that the ball had really not penetrated into the thoracic cavity, and my opinion was sought to corroborate or dispel this favorable view of the case.

I found him sitting up in bed, his back resting on pillows as a support, apparently at ease, and conversing with numerous acquaintances, who had come to visit him. His countenance exhibited no expression of anxiety, and he answered placidly and without effort the questions I put to him. His pulse was 80 in the minute, the respiration easy, the surface of the body normal in temperature and moist. The stethoscope revealed the existence of no difficulty in the respiratory passages, and the normal *Tick Tac* of the heart beat with healthy precision. There were no signs of inflammation or of effusion into the pericardium.

With such freedom from morbid symptoms, I was disposed to concur with his medical adviser in auguring favorably of the case; for although it might be inferred from the external character of the wound, that the ball had passed somewhere into the cavity of the chest, it was not impossible that it had become lodged in some position, where it remained innocuous.

The previous treatment had been gently antiphlogistic; mild aperients, diaphoretics, acidulated drinks and low diet. The consultation resulted in a continuation of a similar mode of treatment, with the injunction, that he should be kept in a state of absolute bodily rest, and free from every cause of mental excitement, as I felt far from certain that he had not sustained mortal injury.

The symptoms in Poole's case illustrate in a remarkable degree some of the peculiarities of wounds of the heart, and also the assertion made by Hervey, that the heart is not very sensible. I am informed by Dr. Putnam, who saw him at one o'clock on the morning of the 25th, about fifteen minutes after the wound was received, that the patient was at first nearly pulseless, was insensible, and that respiration was performed with great difficulty. In this condition, laboring also under the ordinary signs of shock to the system from a gun shot wound, he continued for about four hours, before any signs of reaction were manifested. Vomiting now occurred; this was followed by increased action of the heart, and sensibility gradually returned.

During the same day (25th) he continued improving, and on the evening visit, the pulse beat 84. The skin was moist and natural, tongue healthy, with no unfavorable symptoms otherwise. No external hæmorrhage had occurred from the wound, nor had any evidences of internal hæmorrhage been evinced by vomiting or expectoration.

Monday, 26th. The wound was examined more particularly, and no traces of the bullet could be found, or any special indications manifested of its presence in the cavity of the thorax. Symptoms about the same.

Tuesday, 27th. I saw the patient for the first time, and found him in the favorable condition already stated.

Wednesday, 28th. Complained of slight headache, pulse 86, bowels not having been moved, a gentle aperient was ordered, by which the pain in the head was relieved. At times the patient had complained of transient and slight pain about the region of the heart.

Thursday, March 1st. Was called in to see patient a second time. Had slept well; pulse 80; respiration natural; appetite good; skin moist; action of the heart natural. He stated that he felt no pain or unpleasant symptom except weakness, remarking, however, that he felt well enough to go out.

Friday, March 2d. The patient perfectly comfortable; pulse 82.

Saturday, March 3d. Patient so well that, upon visiting him, for the third time, by request, he was found receiving his friends, and, contrary to previous injunctions, conversing freely with them. Enjoined repose.

Sunday, March 4th. No untoward sign connected with either the functions of circulation or respiration. During the day, he received, against positive orders, the visits of more than a hundred people, with whom he conversed. His own statement was that he felt quite well.

Monday, March 5th. Dr. Putnam was sent for early in the morning. At eight o'clock, A. M., the patient was found in a high state of irritability; pulse 120; skin hot and dry, and complains of pain generally; respiration troubled and more frequent. An aperient was ordered, by which the symptoms were much alleviated.

Tuesday, 6th. Was again requested to see patient. Pulse 100; countenance anxious; the adnata tinged yellow; complained of debility, but said he had no pain about the heart; signs of effusion.

Wednesday, 7th March. Passed a restless night, notwithstanding the administration of an anodyne; pulse 120; countenance more anxious; respiration much troubled; inability to remain in the recumbent posture; symptoms gradually becoming more grave.

At two o'clock, A. M., Thursday morning, his attending physician was sent for. The patient was now rapidly sinking; pulse almost imperceptible, and with difficulty counted; respiration short, frequent, and difficult; extremities cold; countenance pallid and hippocratic. From this time he continued to sink, and expired, without a struggle, at five o'clock.

*Autopsy* seven hours after death.—The body was in a state of perfect preservation, and showed a powerful and well-developed organization.

The surface of the body presented three orifices of gun-shot wounds: two on the external side of the right thigh, a short distance above the patella, by which, apparently, a ball had made its entrance and exit, respectively; and one on the anterior aspect of the chest, three-quarters of an inch to the left of the median line, and about half an inch below a line drawn across the chest, from one nipple to the other. The examination revealed that all the organs of the body were in a healthy condition.

The sternum and cartilages of the ribs having been partially elevated, a bullet probe could be passed without difficulty, slanting from right to left, through the wall of the thorax, at the place of junction of the cartilages of the fifth or sixth ribs with the margin of the sternum.

The sternum being completely elevated, the pericardium was seen to be much distended, and on its surface, in continuation with the external wound, was observed a rough spot, which proved to be an opening into the cavity of the pericardium, thinly closed by the exudation ot plastic material.

The right and left cavities of the pleura were free from effusion, and the lung on each side was in a sound condition. The pericardium was found filled with serous fluid, tinged with blood, and was so distended that it encroached very much upon the lungs on both sides. Upon opening the sac of the pericardium, and removing the large quantity of serous fluid, the external surface of the heart and the serous lining of the pericardium were both found to be entirely covered with plastic exudation, presenting all over signs of high inflammatory action. A cursory examination of the heart in position did not disclose the presence of any foreign body. It was afterwards taken out, and upon a careful examination, a bullet one inch in circumference, was found enveloped in a delicate cyst, and imbedded, to the depth of a quarter of an inch, in the muscular tissue of the septum, between the right and left ventricles, about midway between the apex of the heart and the base of the ventricles. Its locality was only indicated by the sense of touch, for as the wound had entirely cicatrized, there was no outward visible sign of its presence. Obviously the cause of death was inflammation of the pericardium and heart and its results.

This case is one to be added to the few already on authentic record showing that penetrating wound of the heart are not always immediately mortal.

It has, moreover, peculiar features which will render it remarkable in the annals of surgical pathology.

Several cases are mentioned in which patients have survived one or more days the effects of penetrating and non-penetrating wounds of the heart, inflicted by cutting instruments, and also of non-penetrating wounds inflicted by *gun-shot*.

But the peculiarity of this case is, that although the wound was a penetrating gun-shot wound, leaving the ball deeply buried in the tissue of the heart, the patient survived for a period of time so long as to encourage the hope of recovery.

This position of the ball discriminates the case from that mentioned by the French surgeon, Latour, where the ball had not penetrated deeply into the heart, but rested on its surface, partially encroaching upon the muscular wall of the heart and enfolded partly by the pericardium.

The autopsy of this case also revealed, that the wound was not only closed and cicatrized, but that a cyst was in process of formation around the ball.

By this case, also, it is established, that hæmorrhage is not necessarily a consequence of a gun-shot wound of the heart; for the serum found in the pericardium was merely tinged with blood, and there was no coagulum. The absence of hæmorrhage may be accounted for by the conical shape of the ball, and by its direction, two circumstances which favored its passage between the muscular fibres of the superficial layer of the heart, without severing them, and caused it to rest slantingly behind the anterior coronary artery, without wounding it.—*The American Medical Monthly.*

